# Prevalence and correlates of dental service utilisation among a national general adult population sample in Sudan

**DOI:** 10.1186/s12903-021-01422-5

**Published:** 2021-02-11

**Authors:** Supa Pengpid, Karl Peltzer

**Affiliations:** 1 ASEAN Institute for Health Development, Mahidol University, Salaya, Phutthamonthon, Nakhon Pathom Thailand; 2 Department of Research Administration and Development, University of Limpopo, Turfloop, South Africa; 3 Department of Psychology, University of the Free State, Bloemfontein, South Africa

**Keywords:** Adults, Dental service utilisation, Prevalence, Correlates, Sudan

## Abstract

**Background:**

Prompt dental service utilisation (DSU) is needed for the prevention and treatment of oral diseases, and it is therefore important to determine the facilitators and barriers of DSU. There is, however, scarce information available on DSU in Sudan. Therefore, this study aimed to investigate the prevalence and associated factors of DSU in a general population-based survey among 18–69 year-old persons in Sudan.

**Methods:**

Cross-sectional nationally representative data of 7,722 18–69 year-old persons (36 years median age) from the 2016 Sudan Stepwise approach to surveillance (STEPS) survey were analysed. Using questionnaire, anthropometric and biochemical measures, predisposing, enabling and health and lifestyle factors of DSU were assessed. Multinomial logistic regression was conducted to estimate the predictors of DSU (> 12 months and past 12 months, with never DSU as the reference category).

**Results:**

About two-thirds of the participants (64.6%) had never DSU, 22.0% had more than 12-month DSU, and 13.4% had past 12- month DSU. Among those who had ever DSU, the main reason for the last DSU was pain or trouble with teeth, gums, or mouth (66.9%), treatment or follow-up treatment 22.3%, and routine check-up treatment 5.0%. In adjusted multinomial logistic regression analysis, higher education (*p* < 0.001), urban residence (*p* < 0.001), screened for blood pressure (*p* < 0.001), raised total cholesterol (*p* < 0.05), poor SROH (*p* < 0.001), pain in teeth or mouth (*p* < 0.001), and not working because of teeth or mouth (*p* < 0.01) were positively and not knowing their household income (*p* < 0.01), high physical activity (*p* < 0.05), and having 20 or more teeth (*p* < 0.001), were negatively associated with both > 12 months and past 12 months DSU. Higher household income (*p* < 0.001), overweight/obesity (*p* < 0.05), using tooth paste (*p* < 0.001), and difficulty chewing (*p* < 0.001), were positively, and male sex (*p* < 0.01), and teeth cleaning twice or more times a day (*p* < 0.05), were negatively associated with > 12 months or past 12 months DSU.

**Conclusion:**

More than one in ten participants had past 12 months DSU and several factors were detected which could be targeted in intervention activities. Study findings suggest to improve oral health awareness, in particular stressing the relevance of regular dental check-ups, by using different modalities of oral health promotion.

## Introduction

Oral diseases pose a major health burden globally causing pain, discomfort, disfigurement and even death, and are increasing in low- and middle-income countries [1]. “The majority of oral health conditions are: dental caries (tooth decay), periodontal diseases, oral cancers, oral manifestations of HIV, oro-dental trauma, cleft lip and palate, and noma.” [1] “Globally, it is estimated that 2.3 billion people suffer from caries of permanent teeth, and severe periodontal diseases are estimated to affect nearly 10% of the global population.” [1] The prevalence of untreated caries and periodontal disease was high in the general population in Khartoum State, Sudan [2].

Sudan has a population of 45.5 million people, life expectancy at birth is 66.5 years, 35.3% live in urban areas, 60.7% can read and write, and there are 0.26 physicians per 1,000 population [3]. In Sudan, the dentist-to-patient ratio is 1:33,000 [2, 4]. “Relative to the size of the Sudanese population, there are very few dentists and this restricts access to regular dental care. Other factors which influence dental attendance in Sudan include the lack of public funding for oral healthcare and dental insurance schemes to ameliorate the cost of care.” [2]. In an assessment of dental services in urban and rural areas in the Gezira locality in Sudan, a poor provision of dental services in both quantity and quality was found [5]. In Sudan, the health care share of out-of-pocket expenditure is 78.9% (2013) [6]. Households use one 1% of health expenses on dental care [7]. The National Health Insurance Fund (NHIF) covered 43.8% of the Sudanese population at the end of 2016 [8]. People who are not covered by health insurance have to pay user fees in public sector health facilities in Sudan [7]. The “utilization of outpatient dental healthcare is 63% higher for insured people than for the non-insured” in Sudan [7]. The “public sector operates 3,726 family health centers/units, 141 locality hospital and 55 hospitals”, while the private sector, concentrated in major cities, operates 319 health centres and 49 hospitals [7].

According to data from the World Health Survey [9], adults expressing a need for oral health services range from 35% in low-income countries to 60% in lower-middle-income countries. Prompt dental service utilisation (DSU) is needed for the prevention and treatment of oral diseases, and it is therefore important to determine the facilitators and barriers of DSU [10]. However, no national population-based study has been conducted on the prevalence and correlates of DSU in Sudan, a low-income country in north-eastern Africa. In an assessment study on the clinical oral health status among Sudanese adults in Khartoum State, a high prevalence of caries was found, 22.7% had never DSU, 16.7% every two years DSU and 60.6% less than every two years DSU [2]. In a health centre based survey of beneficiaries of Health Insurance Corporation Khartoum State, Sudan (N = 442), 46% had past 12-month DSU [11], and among dental patients in Khartoum (N = 1262), 53.9% has past two years DSU [12],

In a review including studies from 28 countries found a global mean “proportion of individuals regularly/preventively utilizing dental services was 54%.” [13]. In specific countries, the past 12-month dental service utilization (DSU) was 21.4% in 35–44 year-olds and 20.7% in 65–74 year-olds in China [14], in adults is Bejing, 11.3% per year [15], and in Iran 56% (15–64 years) [16]. Some studies have reported the prevalence never-DSU among the general adult population, ranging from 3.3% in Brazil [17] to 73.6% in Nigeria [18], and 86.4% in Indonesia [19]. Furthermore, the national study in Nigeria [18] showed the prevalence of self-reported reasons for DSU, namely, 54.9% for treatment, 24.9% for check-up only, and the remainder for both treatment and check-up, while in two studies in Greece the reasons were 31.7% of the visits were for a regular dental check up [20], and 32.6% reported prevention as the reason for visit [21], and in Khourtum State in Sudan most (> 91%) indicated pain to be the reason for going to the dentist [4].

Predisposing factors of DSU include female sex [10, 12–15, 19], older age [16, 19, 22], decreasing age [10, 15], no age differences [13], higher educational level [14, 16, 19, 22, 23], and higher household income/wealth [14, 16, 19–23]. Enabling factors of DSU include having medical insurance [14, 15, 22, 23], urban residence [10, 19, 23], short distance to dentist [15], and oral health literacy [13, 15].

Health and lifestyle factors of DSU include better general health status [13], having normal weight [20], having non-communicable diseases [10], never smoking [19], low alcohol use [24], being physically active [20], and having a healthier diet [20]. Perceived need factors of DSU include poor self-rated oral health (SROH) [13–16, 22], pain in teeth or gum [19, 25], self-reported mouth ulcers [19], more permanent teeth [25], not edentulous, nor severe tooth loss [13], and eating difficulties due to oral problems [26]. The study aimed to investigate the prevalence and associated factors of DSU in a general population-based survey among 18–69 year-old persons in Sudan.

### Methods

#### Participants and procedures

The sample included 7722 adults (18–69 years, 36 years median; interquartile range: 23–43) that participated in the cross-sectional 2016 Sudan Stepwise approach to surveillance (STEPS) Survey [27, 28]. STEPS focus is on “obtaining population-based data on the established risk factors that determine the major disease burden” [27]. “STEPS surveys collect data at three levels: Step 1- Questionnaire-based assessment includes socio-economic data, data on tobacco and alcohol use, nutritional status, and physical inactivity; Step 2- includes simple physical measurements, such as height, weight, waist circumference, and blood pressure; and Step 3- includes biochemical measurements” [27].”

A nationally representative population-based sample was selected using a multistage sampling approach. The 2016 Sudan STEPS survey was conducted from February to December 2016. The study response rate was 88%; further details and data can be accessed [27, 28].

All methods were performed in accordance with the relevant guidelines and regulations.

All methods were carried out in accordance with the Declaration of Helsinki.

The study protocol was approved by the “national Ethical Committee at the Federal Ministry of Health, Sudan” [27, 28]. Informed consent was obtained from all subjects or, if subjects are under 18, from a parent and/or legal guardian [27, 28].

*Sample size calculation*. In a systematic review and meta-analysis the mean past 12 month DSU was 21.4% [13]. Based on this information, the sample size was calculated with an expected DSU prevalence of 21%, acceptable margin of 5%, confidence level 99.99% and clusters 302; the minimum sample for each cluster is 4, the minimum sample is 1208. In this study we used all 7,722 participants for the analysis.

### Measures

The study used the STEPS Survey questionnaire (see Additional file 1) [27, 28].

*DSU* was assessed with the question,”How long has it been since you last saw a dentist?” Response options were: < 6 months, 6–12 months, > 1 year and < 2 years, > 2 years and < 5 years, ≥ 5 years, and never received dental care [26]. Furthermore, participants were asked, “What was the main reason for your last visit to the dentist?” (Response options ranged from 1 = consultation/advice to 4 = other, see more details in Table 2) [28].

*Predisposing factors* included age, sex, household income, and highest educational level [28].

*Enabling factors* included (1) residence status, (2) health care workers advised to “quit using tobacco or don’t start” in the past three years, (3) ever had blood pressure measured by a health care worker? (4) ever had blood sugar measured? and (5) ever had cholesterol (fat levels in blood) measured) [28].

#### Health and lifestyle factors

Using WHO STEPS standard methodology [27], diabetes was defined as “fasting plasma glucose levels ≥ 7.0 mmol/L, and/or currently taking insulin or oral hypoglycemic drugs,”[27] hypertension (BP) was defined as “systolic BP ≥ 140 mm Hg and/or diastolic BP ≥ 90 mm Hg or currently on antihypertensive medication” (mean of the last two of three readings), raised total cholesterol (TC) (“fasting TC ≥ 5.0 mmol/L or currently on medication for raised cholesterol”), and general overweight/obesity (measured Body Mass Index:”25–29.9 kg/m^2^ overweight and ≥ 30 kg/m^2^ obesity”) [28].

*Oral health-related behaviours* were sourced from two items: 1) “How often do you clean your teeth?” (“1 = never to 7 = twice or more a day”), and 2) “Do you use toothpaste?” [28]

*Other health behaviours* included past and current tobacco smoking, current smokeless tobacco use, daily fruit and vegetable intake [28], and high, moderate, or low physical activity measured with the “Global Physical Activity Questionnaire (GPAQ).” [29]

#### Need factors

*SROH* was sourced from two items, 1) “How would you describe the state of your teeth, and 2) How would you describe the state of your gums?” [28] Poor SROH was defined as “having poor or very poor status of teeth and/or gums, and good oral health as having an average, good,, very good or excellent status of teeth and/or gums”, in line with previous research [30].

*Oral health impact (OHI)* was sourced from three items, “Difficulty in chewing foods in the past 12 months?” embarrassment about the appearance of teeth in the past 12 months, and “Days not at work because of teeth or mouth?” (“Yes/No”) [28].

*Physical symptoms* were sourced from the item, “During the past 12 months, did your teeth or mouth cause any pain or discomfort?” (“Yes/No”) [28].

*Self-reported number of teeth* was measured with the question “How many natural teeth do you have?” Response options were: no natural teeth, 1–9, 10–19 and ≥ 20 teeth [28].

### Data analysis

Statistical procedures were conducted with STATA software version 15.0 (Stata Corporation, College Station, Texas, USA), taking into account the multistage sampling design and weighting of the data [28]. Multinomial logistic regression was conducted to estimate the predictors of DSU (> 12 months and past 12 months, with never DSU as the reference category). Variables significant in the simple model were subsequently included in the multivariable logistic model. Missing values were not included in the analysis and *p* < 0.05 was seen as significant.

## Results

### Sample and DSU characteristics

In all, 7,722 persons (31 years median age, IQR: 23–43, range 18–69 years) were included in the study sample, 35.1% were males and 64.9% were females, 51.5% had a household income (≤ 1000 Sudanese pounds), and 33.8% could not read and write. The majority (62.9%) of the participants lived in rural locations, 8.5% had been advised not to smoke, 39.0% had ever their blood pressure, 14.6% glucose, and 5.2% their cholesterol measured. Regarding health and lifestyle factors, 28.3% of the sample had overweight/obesity, 5.9% had diabetes, 13.7% raised total cholesterol, and 31.5% hypertension. In terms of health behaviour.

63.2% cleaned their teeth twice or more times a day, 97.5% used toothpaste, 9.6% were currently using smoking tobacco, 7.9% smokeless tobacco use, 5.3% had five or more fruit and vegetable servings a day, and 55.1% had high physicl activity. In terms of need factors, 20.2% had poor SROH, 23.1% had pain in teeth or mouth, 10.8% had less than 20 teeth, 17.5% experienced difficulty in chewing foods in the past 12 months, 3.5% were embarrassed about the appearance of their teeth in the past 12 months, and 6.1% were not at work because of teeth or mouth in the past 12 months. About two-thirds of the participants (64.6%) had never DSU, 22.0% had more than 12-month DSU, and 13.4% had past 12- month DSU (see Table 1).

**Table 1:** Sample and dental service utilisation characteristics among 18–69 year-old persons in Sudan, 2016

Variable	Sample	Dental service utilisation		
		Never	> 12 months	≤ 12 months
	N (%)	%	%	%
All	7722	64.6	22.0	13.4
**Predisposing factors**				
Age group (in years)				
18–34	3454 (57.7)	72.9	15.7	11.4
35–49	2474 (26.5)	56.4	28.0	15.5
50–69	1794 (15.8)	48.0	34.7	17.3
Sex				
Female	5016 (64.9)	57.5	25.9	16.6
Male	2707 (35.1)	70.6	18.7	10.7
Education				
Cannot write nor read	3272 (33.8)	76.2	15.6	8.2
Primary or less	2481 (34.2)	69.1	18.7	12.2
More than primary	1952 (32.0)	47.6	32.2	20.2
Household income				
≤ 500	1326 (18.6)	75.3	14.0	10.7
≤ 1000	2632 (32.9)	68.0	20.2	11.8
≤ 2000	1949 (24.3)	51.3	31.9	16.8
> 2000	727 (10.4)	37.1	37.6	25.3
Do not know	1025 (13.7)	84.8	37.6	6.7
**Enabling factors**				
Urban residence	2593 (37.1)	46.6	32.8	20.6
Advised to not smoke by health care worker	609 (8.5)	61.4	19.6	19.0
Ever screened for blood pressure	3446 (39.0)	43.9	34.4	21.8
Ever screened for glucose	1397 (14.6)	33.2	39.2	27.2
Ever screened for cholesterol	457 (5.2)	35.0	38.8	26.2
**Health and lifestyle factors**				
Hypertension	2710 (31.5)	57.1	27.6	15.3
Raised total cholesterol	1229 (13.7)	44.2	34.4	21.3
Diabetes	515 (5.9)	41.1	39.4	19.5
Overweight/obesity	2455 (28.3)	46.8	33.2	20.0
Teeth cleaning (≥ twice/day)	4775 (63.2)	64.8	22.0	13.2
Uses toothpaste	6731 (87.5)	61.8	23.6	14.6
Tobacco smoking				
Never	6964 (85.4)	65.1	21.6	13.2
Past	295 (5.0)	56.8	27.5	15.7
Current	463 (9.6)	64.2	22.1	13.7
Current smokeless tobacco use	458 (7.9)	64.5	22.6	12.9
Fruit/vegetable intake (< 5 servings/day)	7220 (94.7)	65.0	21.8	13.3
Physical activity				
Low	1827 (21.2)	57.2	28.0	14.8
Moderate	2023 (23.7)	59.7	24.5	15.8
High	3771 (55.1)	69.2	18.9	11.9
**Need factors**				
Poor self-rated oral health	1766 (20.2)	38.5	34.4	27.2
Pain in teeth/mouth (past year)	2060 (23.1)	33.2	27.9	38.9
Teeth (≥ 20)	6533 (89.2)	66.0	21.2	12.8
OHI chewing	1594 (17.5)	35.9	28.6	35.5
OHI embarrassed	330 (3.5)	35.9	28.8	35.3
OHI not work	469 (6.1)	44.4	27.0	28.6

OHI = Oral Health Impact

Among those who had ever DSU, the main reason for the last DSU was pain or trouble with teeth, gums, or mouth (66.9%), treatment or follow-up treatment 22.3%, and routine check-up treatment 5.0% (see Table 2).

**Table 2:** Main reason for last dental visit

Main reason for last dental visit	All	Male	Female
	%	%	%
Consultation/advice	4.5	6.6	2.7
Pain or trouble with teeth, gums or mouth	66.9	65.9	67.8
Treatment/Follow-up treatment	22.3	19.7	24.8
Routine check-up treatment	5.0	6.5	3.7
Other	1.0	1.3	1.0

### Multinomial logistic regression with DSU

In adjusted multinomial logistic regression analysis, higher education, urban residence, screened for blood pressure, raised total cholesterol, poor SROH, pain in teeth or mouth, and not working because of teeth or mouth were positively and not knowing their household income, high physical activity and having 20 or more teeth were negatively associated with both > 12 months and past 12 months DSU. Higher household income, overweight/obesity, using tooth paste, and difficulty chewing were positively, and male sex and tooth cleaning twice or more times a day were negatively associated with > 12 months or past 12 months DSU. In addition, in unadjusted analysis, having been advised not to smoke, ever screened for glucose, ever screened for cholesterol, hypertension, diabetes, past tobacco smoking, and embarrassment because of the appearance of their teeth were positively associated with > 12 months and/or past 12 months DSU (see Table 3).

**Table 3:** Associations with > 12 months and past 12 months dental service utilisation (DSU) (with never DSU as reference category)

Variable	Dental service utilisation		Dental service utilisation	
	> 12 months	≤ 12 months	> 12 months	≤ 12 months
	CRRR (95% CI)	CRRR (95% CI)	ARRR (95% CI)	ARRR (95% CI)
**Predisposing factors**				
Age group (in years)				
18–34	1 (Reference)	1 (Reference)	1 (Reference)	1 (Reference)
35–49	2.31 (1.94, 2.75)***	1.76 (1.43, 2.17)***	1.89 (1.52, 2.36)***	1.00 (0.77, 1.32)
50–69	3.36 (2.77, 4.08)***	2.30 (1.84, 2.89)***	2.51 (1.90, 3.30)***	1.28 (0.92, 1.78)
Sex				
Female	1 (Reference)	1 (Reference)	1 (Reference)	1 (Reference)
Male	0.59 (0.49, 0.70)***	0.53 (0.39, 0.52)***	0.73 (0.58, 0.93)**	0.77 (0.57, 1.03)
Education				
Cannot write nor read	1 (Reference)	1 (Reference)	1 (Reference)	1 (Reference)
Primary or less	1.32 (1.09, 1.60)**	1.63 (1.29, 2.06)***	1.47 (1.16, 1.87)**	2.19 (1.62. 2.95)***
More than primary	3.30 (2.54, 4.30)***	3.93 (1.97, 5.22)***	2.45 (1.81, 3.32)***	3.37 (2.40, 4.74)***
Household income				
≤ 500	1 (Reference)	1 (Reference)	1 (Reference)	1 (Reference)
≤ 1000	1.60 (1.24, 2.06)***	1.22 (0.91, 1.63)	1.04 (0.79, 1.38)	0.63 (0.43, 0.93)*
≤ 2000	3.34 (2.47, 4.52)***	2.30 (1.70, 3.11)***	1.82 (1.30, 2.55)***	0.88 (0.58, 1.34)
> 2000	5.45 (3.60, 8.24)***	4.78 (3.17, 7.23)***	2.25 (1.47, 3.43)***	1.51 (0.85, 2.67)
Do not know	0.54 (0.38, 0.76)***	0.56 (0.36, 0.86)**	0.50 (0.33, 0.75)***	0.41 (0.23, 0.73)**
**Enabling factors**				
Urban residence	3.40 (2.53, 4.56)***	3.63 (2.72, 4.85)***	1.93 (1.47, 2.55)***	1.71 (1.23, 2.37)***
Advised to not smoke by health care worker	0.93 (0.67, 1.30)	1.56 (1.08, 2.27)*	0.73 (0.51, 1.04)	0.96 (0.59, 1.57)
Ever screened for blood pressure	4.09 (3.34, 5.02)***	4.48 (3.54, 5.67)***	2.04 (1.62, 2.57)***	2.09 (1.52, 2.86)***
Ever screened for glucose	4.40 (3.58, 5.41)***	5.18 (4.11, 6.54)***	1.20 (0.91, 1.59)	1.35 (1.00, 1.84)
Ever screened for cholesterol	3.49 (2.46, 4.96)***	3.90 (2.76, 5.52)***	1.06 (0.72, 1.54)	1.24 (0.80, 1.92)
**Health and lifestyle factors**				
Hypertension	1.68 (1.44, 1.97)***	1.44 (1.17, 1.75)**	1.07 (0.87, 1.30)	0.98 (0.74, 1.31)
Raised total cholesterol	2.63 (2.13, 3.23)***	2.66 (2.04, 3.49)***	1.32 (1.02, 1.70)*	1.49 (1.05, 2.12)*
Diabetes	3.04 (2.22, 4.16)***	2.43 (1.65, 3.57)***	1.14 (0.79, 1.65)	0.95 (0.59, 1.54)
Overweight/obesity	2.79 (2.33, 3.35)***	2.73 (2.21, 3.38)***	1.27 (1.06, 1.55)*	1.32 (0.97, 1.78)
Teeth cleaning (≥ twice/day)	0.48 (0.35, 0.64)***	0.42 (0.31, 0.58)***	0.75 (0.60, 0.94)*	0.78 (0.56, 1.08)
Uses toothpaste	3.09 (0.25, 4.24)***	3.76 (2.34, 6.03)***	1.88 (1.18, 2.76)***	1.62 (0.94, 2.78)
Tobacco smoking				
Never	1 (Reference)	1 (Reference)	1 (Reference)	1 (Reference)
Past	1.46 (1.04, 2.04)*	1.36 (0.94, 1.96)	0.86 (0.53, 1.39)	
Current	1.04 (0.78, 1.39)	1.05 (0.72, 1.53)	1.02 (0.67, 1.57)	
Current smokeless tobacco use	1.03 (0.74, 1.43)	0.96 (0.65, 1.42)	–	–
Fruit/vegetable intake (< 5 servings/day)	0.63 (0.45, 0.90)**	0.64 (0.42, 0.96)*	1.13 (0.75, 1.70)	1.16 (0.65, 2.06)
Physical activity				
Low	1 (Reference)	1 (Reference)	1 (Reference)	1 (Reference)
Moderate	0.84 (0.66, 1.07)	1.03 (0.78, 1.35)	0.80 (0.59, 1.09)	0.78 (0.55, 1.11)
High	0.56 (0.45, 0.69)***	0.67 (0.51, 0.88)**	0.73 (0.56, 0.95)*	0.66 (0.46, 0.95)*
**Need factors**				
Poor self-rated oral health	3.39 (2.82, 4.08)***	5.29 (4.32, 6.49)***	1.71 (1.35, 2.17)***	1.71 (1.29, 2.27)***
Pain in teeth/mouth (past year)	3.08 (2.51, 3.77)***	15.08 (11.79, 19.28)***	1.88 (1.41, 2.51)***	7.63 (5.58, 10.45)***
Teeth (≥ 20)	0.48 (0.35, 0.64)***	0.42 (0.31, 0.58)***	0.53 (0.38, 0.73)***	0.48 (0.32, 0.71)***
OHI chewing	2.74 (2.23, 3.37)***	8.04 (6.44, 10.06)***	1.26 (0.92, 1.73)	1.95 (1.38, 2.75)***
OHI embarrassed	2.43 (1.65, 3.56)***	5.12 (3.48, 7.55)***	0.96 (0.61, 1.53)	1.21 (0.73, 1.98)
OHI not work	1.85 (1.37, 2.50)***	3.41 (2.44, 4.78)***	1.50 (1.05, 2.13)*	2.06 (1.31, 3.23)**

OHI = Oral Health Impact; CRRR = Crude Relative Risk Ratios; ARRR = Adjusted Relative Risk Ratios

***P < 0.001; **P < 0.01; *P < 0.05

## Discussion

In this nationally representative general adult population in Sudan, past 12-month DSU (13.4%) was higher than in Beijing (11.3%) [15], but lower than in a population-based study among Sudanese adults in Khartoum State (16.7% every two years DSU) [2], in a health centre based survey in Khartoum State (46% had past 12-month DSU) [11], and among dental patients in Khartoum (53.9% has past two years DSU) [12], and lower than in a global study in 28 countries (54%) [13]. The prevalence of never DSU in this study (64.6%) was higher than in Brazil (3.3%) [17], and in a previous study in Khartoum State (22.7%) [2], but lower than in Nigeria (73.6%) [18] and Indonesia (86.4%) [19]. The prevalence of DSU in Khartoum State may be higher than in the rest of the country because of better access to dental services in the Khartoum area. Consistent with some studies in Greece, Nigeria, and Khartoum State, Sudan [2, 18, 20, 21], the main reason for DSU was treatment rather than dental check-up. Khalifa et al. [2] note the “apparent lack of restorative or preventive dental care, as shown by the filled component F (0.2%) and treatment is limited to pain relief or emergency care by tooth extraction” in Khartoum State, Sudan [2]. This result highlights the lack of awareness about the importance of preventive DSU and possibly the lack of physical access to dental care services. By all means, DSU needs to be improved in Sudan.

In terms of predisposing factors, DSU increased with age, higher education, higher income, and among women. Similar results were found in some previous studies [10, 13, 14, 16, 19–23]. Older persons tend to have more dental problems, and since most DSU were sequel to pain or trouble with teeth or mouth, it may explain the higher proportion of older adults with DSU [18]. Individuals with higher education may have greater awareness of oral health risks and the importance of DSU [10, 16]. Higher income may increase the ability to access DSU [15]. Women may focus more on their oral health than men, and may thus have more oral health behaviours, including DSU [19].

Regarding enabling factors, this study showed that urban residence and previous health screening (for blood pressure, glucose, and/or cholesterol) increased the odds for DSU. One possible way of increasing DSU is the integration of oral health into the prevention and control of non-commuicable diseases within the framework of enhanced primary health care, such as blood pressure or glucose screening, in Sudan [31].

In terms of health factors and consistent with some studies [10], this study showed that raised total cholesterol, overweight or obesity, and in unadjusted analysis, hypertension and diabetes were associated with DSU. It is possible that non-communicable diseases share lifestyle risk factors with oral diseases, which can increase poor oral health subsequently leading to more DSU [10]. While some previous studies found a positive association between health behaviours and DSU [19, 20, 24], this study only found such associations with using tooth paste and in unadjusted analysis with adequate fruit and vegetable consumption. Contrary to expectations [20], adequate tooth cleaning and high physical activity were negatively associated with DSU. It is possible that the highly physically active population belongs to a lower socioecomic group decreasing the access to DSU.

Regarding perceived need factors, consistent with a number of previous research studies [14–16, 19, 22, 25], this study found a positive association between poor SROH, pain in teeth or mouth, oral health impact (difficulty chewing, and not working) and DSU. Unlike some previous research [5], this study did not find a significant association between nontooth loss and DSU.

The identification of determinants and potential barriers of DSU in the general population in Sudan has dental public health implications. Oral health promotion activities can be targeted at identified barriers of DSU. New and current activities should be reinforced for the active inclusion of groups with less access to DSU, including younger age groups and rural residents, those with lower education, those who have never screened for blood pressure, those not using tooth paste, and those not experiencing poor oral health. Study findings suggest to improve oral health awareness, in particular stressing the relevance of regular dental check-ups, by using different modalities of oral health promotion [14].

The study limitations included that this investigation was limited due to the self-report of data and the cross-sectional survey design. An additional limitation was that some relevant variables for DSU, such as oral examination, medical insurance coverage, oral health literacy, unmet treatment needs, and supportive family structure were not assessed in the 2016 Sudan STEPS survey, and should be incorporated in the future.

## Conclusion

This investigation showed among a nationally representative population of 18 to 69 years in Sudan that more than one in ten participants had past 12 months DSU. Several associated factors for > 12 months and/or past 12 months DSU were identified, including female sex, higher education, higher household income, urban residence, screening for blood pressure, raised total cholesterol, overweight/obesity, tooth cleaning less than twice a day, using tooth paste, having less than 20 teeth, difficulty chewing, poor SROH, pain in teeth or mouth, and not working because of teeth or mouth, which can be targeted in improving DSU. Moreover, oral health promotion should stress the importance of preventive DSU.

## Supplementary Information

**Additional file 1**.

## Data Availability

“The data for the current study are publicly available at the World Health Organization NCD Microdata Repository (URL: https://extranet.who.int/ncdsmicrodata/index.php/catalog).”

## Abbreviations

DSU: Dental service utilization
IQR: Interquartile range
SROH: Self-rated oral health

## References

[1] World Health Organization. Factsheet, Oral health, 2020. URL: https://www.who.int/news-room/fact-sheets/detail/oral-health. Accessed 20 Oct 2020.

[2] Khalifa N, Allen PF, Abu-bakr NH, Abdel-Rahman ME, Abdelghafar KO (2012). A survey of oral health in a Sudanese population. BMC Oral Health.

[3] World Factbook. Sudan. https://www.cia.gov/library/publications/the-world-factbook/geos/su.html

[4] FDI World Dental Federation 2015 The Challenge of Oral Disease: a call for global action. The Oral Health Atlas. 2nd ed. Geneva: FDI World Dental Federation; 2015. URL: https://www.fdiworlddental.org/sites/default/files/media/documents/complete_oh_atlas.pdf. Accessed 3 Sept 2020.

[5] Yousif MAE, Miskeen E (2009). Dental health service in Gezira Locality. Sudanese J Public Health.

[6] World Health Organization (WHO) Regional Office for the Eastern Mediterranean. Sudan health profile 2015. URL: https://rho.emro.who.int/sites/default/files/Profiles-briefs-files/EMROPUB_EN_19610-SUD.pdf. Accessed 20 Jan 2021

[7] Sudan Federal Ministry of Health. Health System Financing Review Report May 2014. URL: file:///C:/Users/user/Downloads/HealthSystemFinancingReviewReport.20141.pdf (20 Jan 21).

[8] The World Bank. Moving toward Universal Health Coverage, Sudan. URL: http://documents1.worldbank.org/curated/en/929661513159699256/pdf/BRI-Moving-Toward-UHC-series-PUBLIC-WorldBank-UHC-Sudan-FINAL-Nov30.pdf. Accessed 20 Jan 2021.

[9] Hosseinpoor AR, Itani L, Petersen PE (2012). Socio-economic inequality in oral healthcare coverage: results from the World Health Survey. J Dent Res.

[10] Šiljak S, Janković J, Marinković J, Erić M, Janevic T, Janković S (2019). Dental service utilisation among adults in a European developing country: findings from a national health survey. Int Dent J.

[11] MAE Ajeeb, AM Mudawi, NM Nurelhuda. Utilization of Dental Services among Beneficiaries of Health Insurance Corporation Khartoum State East African Scholars J Med Sci. 2019;2(1): 5–11. http://khartoumspace.uofk.edu/123456789/17169

[12] Nasir EF, Astrøm AN, David J, Ali RW (2009). Utilization of dental health care services in context of the HIV epidemic- a cross-sectional study of dental patients in the Sudan. BMC Oral Health.

[13] Reda SM, Krois J, Reda SF, Thomson WM, Schwendicke F (2018). The impact of demographic, health-related, and social factors on dental services utilization: systematic review and meta-analysis. J Dent.

[14] Xu M, Cheng M, Gao X, Wu H, Ding M, Zhang C, Wang X, Feng X, Tai B, Hu D, Lin H, Wang B, Wang C, Zheng S, Liu X, Rong W, Wang W, Xu T, Si Y (2020). Factors associated with oral health service utilization among adults and older adults in China, 2015–2016. Community Dent Oral Epidemiol.

[15] Yuan C, Zhu L, Li YL, Liu M, Si Y, Zhang F (2011). Oral health services utilization and influencing factors in downtown community residents older than 15 years in Beijing. Zhonghua Kou Qiang Yi Xue Za Zhi.

[16] Bahramian H, Mohebbi SZ, Khami MR, Asadi-Lari M, Shamshiri AR, Hessari H (2015). Psychosocial determinants of dental service utilization among adults: results from a population-based survey (Urban HEART-2) in Tehran, Iran. Eur J Dent.

[17] Herkrath FJ, Vettore MV, Werneck GL (2020). Utilisation of dental services by Brazilian adults in rural and urban areas: a multi-group structural equation analysis using the Andersen behavioural model. BMC Public Health.

[18] Olusile AO, Adeniyi AA, Orebanjo O (2014). Self-rated oral health status, oral health service utilization, and oral hygiene practices among adult Nigerians. BMC Oral Health.

[19] Santoso CMA, Bramantoro T, Nguyen MC, Bagoly Z, Nagy A (2020). Factors affecting dental service utilisation in Indonesia: a population-based multilevel analysis. Int J Environ Res Public Health.

[20] Koletsi-Kounari H, Tzavara C, Tountas Y (2011). Health-related lifestyle behaviours, socio-demographic characteristics and use of dental health services in Greek adults. Community Dent Health.

[21] Pavi E, Karampli E, Zavras D, Dardavesis T, Kyriopoulos J (2010). Social determinants of dental health services utilisation of Greek adults. Community Dent Health.

[22] Rezaei S, Woldemichael A, Zandian H, Homaie Rad E, Veisi N, Karami MB (2018). Dental health-care service utilisation and its determinants in West Iran: a cross-sectional study. Int Dent J.

[23] Reda SF, Reda SM, Thomson WM, Schwendicke F (2018). Inequality in utilization of dental services: a systematic review and meta-analysis. Am J Public Health.

[24] Slack-Smith LM, Mills CR, Bulsara MK, O'Grady MJ (2007). Demographic, health and lifestyle factors associated with dental service attendance by young adults. Aust Dent J.

[25] Pinto Rda S, Matos DL, de Loyola Filho AI (2012). Características associadas ao uso de serviços odontológicos públicos pela população adulta brasileira. Cien Saude Colet.

[26] Herkrath FJ, Vettore MV, Werneck GL (2018). Contextual and individual factors associated with dental services utilisation by Brazilian adults: a multilevel analysis. PLoS ONE.

[27] World Health Organization (WHO) (2018). STEPwise approach to surveillance (STEPS). URL: https://www.who.int/ncds/surveillance/steps/en/. Accessed 3 April 2020.

[28] Federal Ministry of Health, Sudan. Sudan STEPwise survey for non-communicable diseases risk factors 2016 report. https://extranet.who.int/ncdsmicrodata/index.php/catalog/438. Accessed 5 Sept 2020.

[29] World Health Organization (WHO) (2012). Global physical activity questionnaire (GPAQ) analysis guide.

[30] Pengpid S, Peltzer K (2019). Self-rated oral health status and social and health determinants among community dwelling adults in Kenya. Afr Health Sci.

[31] World Health Organization. Regional Office for Africa. Promoting oral health in Africa: prevention and control of oral diseases and noma as part of essential noncommunicable disease interventions, 2016. https://www.who.int/publications/i/item/promoting-oral-health-in-africa-prevention-and-control-of-oral-diseases-and-noma-as-part-of-essential-noncommunicable-disease-interventions

