# Correction to: Prevalence and correlates of dental service utilisation among a national general adult population sample in Sudan

**DOI:** 10.1186/s12903-022-02333-9

**Published:** 2022-07-18

**Authors:** Supa Pengpid, Karl Peltzer

**Affiliations:** 1 ASEAN Institute for Health Development, Mahidol University, Salaya, Phutthamonthon, Nakhon Pathom, Thailand; 2 Department of Research Administration and Development, University of Limpopo, Turfoop, South Africa; 3 Department of Psychology, University of the Free State, Bloemfontein, South Africa

## Correction to: BMC Oral Health (2021) 21:61 10.1186/s12903-021-01422-5

In this article, the Acknowledgement section was incorrectly published as “The data source, the World Health Organization NCD Microdata Repository (URL: https://extranet.who.int/ncdsmicrodata/index.php/catalog), is hereby acknowledged.”

The correct Acknowledgement should read as “This paper uses data from the 2016 Sudan STEPS survey, implemented by the Ministry of Health with the support of the World Health Organization.”

