# Supplementary material for: Prevalence and correlates of dental service utilisation among a national general adult population sample in Sudan
Source: BMC Oral Health. 2021 Feb 11;21:61. doi: 10.1186/s12903-021-01422-5 (PMC7879665; doi:10.1186/s12903-021-01422-5)
Supplement: Supplementary file 1 — Additional file 1. [file 12903_2021_1422_MOESM1_ESM.pdf]

WHO STEPS Instrument

(Core and Expanded)

The WHO STEPwise approach to noncommunicable disease risk factor surveillance (STEPS)

World Health Organization
20 Avenue Appia, 1211 Geneva 27, Switzerland

For further information: www.who.int/chp/steps

World Health Organization

STEPS Instrument

Overview

Introduction

This is the generic STEPS Instrument which sites/countries will use to develop their tailored instrument. It contains the:

- CORE items (unshaded boxes)
- EXPANDED items (shaded boxes).

Core Items

The Core items for each section ask questions required to calculate basic variables. For example:

- current daily smokers
- mean BMI.

Note: All the core questions should be asked, removing core questions will impact the analysis.

Expanded items

The Expanded items for each section ask more detailed information. Examples include:

- use of smokeless tobacco
- sedentary behaviour.

Guide to the columns

The table below is a brief guide to each of the columns in the Instrument.

Column	Description	Site Tailoring
Question	Each question is to be read to the participants	• Select sections to use.<br>• Add expanded and optional questions as desired.
Response	This column lists the available response options which the interviewer will be circling or filling in the text boxes. The skip instructions are shown on the right hand side of the responses and should be carefully followed during interviews.	• Add site specific responses for demographic responses (e.g. C6).<br>• Change skip question identifiers where necessary.
Code	The column is designed to match data from the instrument into the data entry tool, data analysis syntax, data book, and fact sheet.	This should never be changed or removed. The code is used as a general identifier for the data entry and analysis.

Participant Identification Number |_|_|_| |_|_|_| |_|_|_|

WHO STEPS Instrument
for Noncommunicable Disease Risk Factor Surveillance

<insert country/site name>

Survey Information

Location and Date	Response	Code
Cluster/Centre/Village ID	|_|_|_|_|_|_|	I1
Cluster/Centre/Village name		I2
Interviewer ID	|_|_|_|	I3
Date of completion of the instrument	|_|_| dd |_|_| mm |_|_|_|_| year	I4

Consent, Interview Language and Name	Response	Code
Consent has been read and obtained	Yes 1<br>No 2 **If NO, END**	I5
Interview Language *[Insert Language]*	English 1<br>*[Add others]* 2<br>*[Add others]* 3<br>*[Add others]* 4	I6
Time of interview<br>(24 hour clock)	|_|_| : |_|_| hrs mins	I7
Family Surname		I8
First Name		I9
**Additional Information that may be helpful**		
Contact phone number where possible		I10

Step 1 Demographic Information

CORE: Demographic Information

Question	Response	Code
Sex (*Record Male / Female as observed)*	Male 1<br>Female 2	C1
What is your date of birth?<br>*Don't Know 77 77 7777*	└─┴─┘ └─┴─┘ └─┴─┴─┴─┘ dd mm year *If known, Go to C4*	C2
How old are you?	Years └─┴─┘	C3
In total, how many years have you spent at school and in full-time study (excluding pre-school)?	Years └─┴─┘	C4

EXPANDED: Demographic Information

What is the **highest level of education** you have completed?<br>*[INSERT COUNTRY-SPECIFIC CATEGORIES]*	No formal schooling 1<br>Less than primary school 2<br>Primary school completed 3<br>Secondary school completed 4<br>High school completed 5<br>College/University completed 6<br>Post graduate degree 7<br>Refused 88	C5
What is your *[insert relevant ethnic group / racial group / cultural subgroup / others]* **background**?	[*Locally defined]* 1<br>[*Locally defined]* 2<br>[*Locally defined]* 3<br>Refused 88	C6
What is your **marital status**?	Never married 1<br>Currently married 2<br>Separated 3<br>Divorced 4<br>Widowed 5<br>Cohabitating 6<br>Refused 88	C7
Which of the following best describes your **main work** status over the past 12 months?<br>*[INSERT COUNTRY-SPECIFIC CATEGORIES]*<br>(*USE SHOWCARD)*	Government employee 1<br>Non-government employee 2<br>Self-employed 3<br>Non-paid 4<br>Student 5<br>Homemaker 6<br>Retired 7<br>Unemployed (able to work) 8<br>Unemployed (unable to work) 9<br>Refused 88	C8
How many people older than 18 years, including yourself, live in your household?	Number of people └─┴─┘	C9

**EXPANDED: Demographic Information,** Continued			
**Question**	**Response**		**Code**
Taking **the past year**, can you tell me what the average earnings of the household have been?<br>*(RECORD ONLY ONE, NOT ALL 3)*	Per week └─┴─┴─┴─┴─┴─┴─┘	*Go to T1*	C10a
	OR per month └─┴─┴─┴─┴─┴─┴─┘	*Go to T1*	C10b
	OR per year └─┴─┴─┴─┴─┴─┴─┘	*Go to T1*	C10c
	Refused 88		C10d
If you don't know the amount, can you give an **estimate** of the annual household income if I read some options to you? Is it *[INSERT QUINTILE VALUES IN LOCAL CURRENCY]*<br><br>*(READ OPTIONS)*	≤ Quintile (Q) 1 1<br>More than Q 1, ≤ Q 2 2<br>More than Q 2, ≤ Q 3 3<br>More than Q 3, ≤ Q 4 4<br>More than Q 4 5<br>Don't Know 77<br>Refused 88		C11

Participant Identification Number └─┴─┴─┘ └─┴─┴─┘ └─┴─┴─┘

Step 1 Behavioural Measurements

CORE: Tobacco Use		
Now I am going to ask you some questions about tobacco use.		
**Question**	**Response**	**Code**
Do you **currently** smoke any **tobacco** products, such as cigarettes, cigars or pipes?<br>*(USE SHOWCARD)*	Yes 1<br>No 2 *If No, go to T8*	T1
Do you currently smoke tobacco products **daily**?	Yes 1<br>No 2	T2
How old were you when you **first started** smoking?	Age (years)<br>Don't know 77 └─┴─┘ *If Known, go to T5a/T5aw*	T3
Do you remember how long ago it was?<br>*(RECORD ONLY 1, NOT ALL 3)*<br>*Don't know 77*	In Years └─┴─┘ *If Known, go to T5a/T5aw*	T4a
	OR in Months └─┴─┘ *If Known, go to T5a/T5aw*	T4b
	OR in Weeks └─┴─┘	T4c
On average, **how many** of the following products do you smoke **each day/week?**<br>*(IF LESS THAN DAILY, RECORD WEEKLY)*<br>*(RECORD FOR EACH TYPE, USE SHOWCARD)*<br>*Don't Know 7777*	DAILY↓ WEEKLY↓	
	Manufactured cigarettes └─┴─┴─┴─┘ └─┴─┴─┴─┘	T5a/T5aw
	Hand-rolled cigarettes └─┴─┴─┴─┘ └─┴─┴─┴─┘	T5b/T5bw
	Pipes full of tobacco └─┴─┴─┴─┘ └─┴─┴─┴─┘	T5c/T5cw
	Cigars, cheroots, cigarillos └─┴─┴─┴─┘ └─┴─┴─┴─┘	T5d/T5dw
	Number of Shisha sessions └─┴─┴─┴─┘ └─┴─┴─┴─┘	T5e/T5ew
	Other └─┴─┴─┴─┘ └─┴─┴─┴─┘<br>*If Other, go to T5other, else go to T6*	T5f/T5fw
	Other (please specify): └─┴─┴─┴─┴─┴─┘	T5other/<br>T5otherw
During the past 12 months, have you tried to **stop smoking**?	Yes 1<br>No 2	T6
During any visit to a doctor or other health worker in the past 12 months, were you advised to quit smoking tobacco?	Yes 1 *If T2=Yes, go to T12; if T2=No, go to T9*<br>No 2 *If T2=Yes, go to T12; if T2=No, go to T9*<br>No visit during the past 12 months 3 *If T2=Yes, go to T12; if T2=No, go to T9*	T7
In the past, did you **ever smoke** any tobacco products?<br>*(USE SHOWCARD)*	Yes 1<br>No 2 *If No, go to T12*	T8
In the past, did you **ever** smoke **daily**?	Yes 1 *If T1=Yes, go to T12, else go to T10*<br>No 2 *If T1=Yes, go to T12, else go to T10*	T9

EXPANDED: Tobacco Use

Question	Response	Code
How old were you when you **stopped** smoking?	Age (years)<br>Don't Know 77 └─┴─┘ *If Known, go to T12*	T10
How **long ago** did you stop smoking?<br>*(RECORD ONLY 1, NOT ALL 3)*<br>*Don't Know 77*	Years ago └─┴─┘ *If Known, go to T12*	T11a
	OR Months ago └─┴─┘ *If Known, go to T12*	T11b
	OR Weeks ago └─┴─┘	T11c
Do you **currently use** any **smokeless tobacco** products such as *[snuff, chewing tobacco, betel]*? *(USE SHOWCARD)*	Yes 1<br>No 2 *If No, go to T15*	T12
Do you **currently use smokeless tobacco** products **daily?**	Yes 1<br>No 2 *If No, go to T14aw*	T13
On average, how many **times a day/week** do you use ….<br>*(IF LESS THAN DAILY, RECORD WEEKLY)*<br>*(RECORD FOR EACH TYPE, USE SHOWCARD)*<br>*Don't Know 7777*	DAILY↓ WEEKLY↓	
	Snuff, by mouth └─┴─┴─┴─┘ └─┴─┴─┴─┘	T14a/ T14aw
	Snuff, by nose └─┴─┴─┴─┘ └─┴─┴─┴─┘	T14b/ T14bw
	Chewing tobacco └─┴─┴─┴─┘ └─┴─┴─┴─┘	T14c/ T14cw
	Betel, quid └─┴─┴─┴─┘ └─┴─┴─┴─┘	T14d/ T14dw
	Other └─┴─┴─┴─┘ └─┴─┴─┴─┘<br>*If Other, go to T14other, if T13=No, go to T16, else go to T17*	T14e/ T14ew
	Other (please specify): └─┴─┴─┴─┴─┴─┘<br>*If T13=No, go to T16, else go to T17*	T14other/ T14otherw
In the **past**, did you **ever use** smokeless tobacco products such as *[snuff, chewing tobacco, or betel]*?	Yes 1<br>No 2 *If No, go to T17*	T15
In the **past**, did you **ever use** smokeless tobacco products such as *[snuff, chewing tobacco, or betel]* **daily**?	Yes 1<br>No 2	T16
During the past 30 days, did someone smoke **in your home**?	Yes 1<br>No 2	T17
During the past 30 days, did someone smoke in closed areas **in your workplace** (in the building, in a work area or a specific office)?	Yes 1<br>No 2<br>Don't work in a closed area 3	T18

CORE: Alcohol Consumption

The next questions ask about the consumption of alcohol.

Question	Response	Code
Have you **ever** consumed any alcohol such as beer, wine, spirits or *[add other local examples]*?<br>*(USE SHOWCARD OR SHOW EXAMPLES)*	Yes 1<br>No 2 *If No, go to A16*	A1
Have you consumed any alcohol within the **past 12 months**?	Yes 1 *If Yes, go to A4*<br>No 2	A2
Have you stopped drinking due to health reasons, such as a negative impact on your health or on the advice of your doctor or other health worker?	Yes 1 *If Yes, go to A16*<br>No 2 *If No, go to A16*	A3
During the past 12 months, **how frequently** have you had at least one standard alcoholic drink?<br>*(READ RESPONSES, USE SHOWCARD)*	Daily 1<br>5-6 days per week 2<br>3-4 days per week 3<br>1-2 days per week 4<br>1-3 days per month 5<br>Less than once a month 6	A4
Have you consumed any alcohol within the **past 30 days**?	Yes 1<br>No 2 *If No, go to A13*	A5
During the past 30 days, on how many **occasions** did you have at least one standard alcoholic drink?	Number<br>Don't know 77 |__|__|	A6
During the past 30 days, when you drank alcohol, how many **standard drinks on average** did you have during one drinking occasion?<br>*(USE SHOWCARD)*	Number<br>Don't know 77 |__|__|	A7
During the past 30 days, what was the **largest number** of standard drinks you had on a single occasion, counting all types of alcoholic drinks together?	Largest number<br>Don't Know 77 |__|__|	A8
During the past 30 days, how many times did you have **six or more** standard drinks in a single drinking occasion?	Number of times<br>Don't Know 77 |__|__|	A9
During each of the **past 7 days**, how many standard drinks did you have each day?<br>*(USE SHOWCARD)*<br>*Don't Know 77*	Monday |__|__|	A10a
	Tuesday |__|__|	A10b
	Wednesday |__|__|	A10c
	Thursday |__|__|	A10d
	Friday |__|__|	A10e
	Saturday |__|__|	A10f
	Sunday |__|__|	A10g

CORE: Alcohol Consumption, continued

I have just asked you about your consumption of alcohol during the past 7 days. The questions were about alcohol in general, while the next questions refer to your consumption of homebrewed alcohol, alcohol brought over the border/from another country, any alcohol not intended for drinking or other untaxed alcohol. Please only think about these types of alcohol when answering the next questions.

Question	Response	Code
During the **past 7 days**, did you consume any **homebrewed** alcohol, any alcohol **brought over the border/from another country**, any alcohol **not intended for drinking** or other **untaxed** alcohol?<br>*[AMEND ACCORDING TO LOCAL CONTEXT]*<br>*(USE SHOWCARD)*	Yes 1<br>No 2 *If No, go to A13*	A11
On average, **how many standard drinks** of the following did you consume **during the past 7 days**?<br>*[INSERT COUNTRY-SPECIFIC EXAMPLES]*<br>*(USE SHOWCARD)*<br>*Don't Know 77*	Homebrewed spirits, e.g. moonshine └─┴─┘	A12a
	Homebrewed beer or wine, e.g. beer, palm or fruit wine └─┴─┘	A12b
	Alcohol brought over the border/from another country └─┴─┘	A12c
	Alcohol not intended for drinking, e.g. alcohol-based medicines, perfumes, after shaves └─┴─┘	A12d
	Other untaxed alcohol in the country └─┴─┘	A12e

EXPANDED: Alcohol Consumption

During the **past 12 months,** how often have you found that you were not able to stop drinking once you had started?	Daily or almost daily 1<br>Weekly 2<br>Monthly 3<br>Less than monthly 4<br>Never 5	A13
During the **past 12 months,** how often have you failed to do what was normally expected from you because of drinking?	Daily or almost daily 1<br>Weekly 2<br>Monthly 3<br>Less than monthly 4<br>Never 5	A14
During the **past 12 months,** how often have you needed a first drink in the morning to get yourself going after a heavy drinking session?	Daily or almost daily 1<br>Weekly 2<br>Monthly 3<br>Less than monthly 4<br>Never 5	A15
During the **past 12 months**, have you had family problems or problems with your partner due to **someone else's** drinking?	Yes, more than monthly 1<br>Yes, monthly 2<br>Yes, several times but less than monthly 3<br>Yes, once or twice 4<br>No 5	A16

CORE: Diet

The next questions ask about the fruits and vegetables that you usually eat. I have a nutrition card here that shows you some examples of local fruits and vegetables. Each picture represents the size of a serving. As you answer these questions please think of a typical week in the last year.

Question	Response	Code
In a typical week, on how many days do you **eat fruit**? *(USE SHOWCARD)*	Number of days<br>Don't Know 77 └─┴─┘ *If Zero days, go to D3*	D1
How many **servings** of fruit do you eat on **one** of those days? (*USE SHOWCARD)*	Number of servings<br>Don't Know 77 └─┴─┘	D2
In a typical week, on how many days do you **eat vegetables**? *(USE SHOWCARD)*	Number of days<br>Don't Know 77 └─┴─┘ *If Zero days, go to D5*	D3
How many **servings** of vegetables do you eat on one of those days? *(USE SHOWCARD)*	Number of servings<br>Don't know 77 └─┴─┘	D4

Dietary salt

With the next questions, we would like to learn more about salt in your diet. Dietary salt includes ordinary table salt, unrefined salt such as sea salt, iodized salt, salty stock cubes and powders, and salty sauces such as soya sauce or fish sauce (see showcard). The following questions are on adding salt to the food right before you eat it, on how food is prepared in your home, on eating processed foods that are high in salt such as *[insert country specific examples]*, and questions on controlling your salt intake. Please answer the questions even if you consider yourself to eat a diet low in salt.

Question	Response	Code
How often do you **add salt or a salty sauce such as soya sauce** to your food right before you eat it or as you are eating it?<br>*(SELECT ONLY ONE)*<br>*(USE SHOWCARD)*	Always 1<br>Often 2<br>Sometimes 3<br>Rarely 4<br>Never 5<br>Don't know 77	D5
How often is **salt, salty seasoning or a salty sauce added** in cooking or preparing foods in your household?	Always 1<br>Often 2<br>Sometimes 3<br>Rarely 4<br>Never 5<br>Don't know 77	D6
How often do you eat **processed food high in salt**? By processed food high in salt, I mean foods that have been altered from their natural state, such as packaged salty snacks, canned salty food including pickles and preserves, salty food prepared at a fast food restaurant, cheese, bacon and processed meat *[add country specific examples].*<br>*[INSERT EXAMPLES]*<br>*(USE SHOWCARD)*	Always 1<br>Often 2<br>Sometimes 3<br>Rarely 4<br>Never 5<br>Don't know 77	D7
**How much salt or salty sauce** do you think you consume?	Far too much 1<br>Too much 2<br>Just the right amount 3<br>Too little 4<br>Far too little 5<br>Don't know 77	D8

EXPANDED: Diet

Question	Response	Code
How important to you is **lowering the salt** in your diet?	Very important 1<br>Somewhat important 2<br>Not at all important 3<br>Don't know 77	D9
Do you think that too much salt or salty sauce in your diet could cause a **health problem**?	Yes 1<br>No 2<br>Don't know 77	D10
Do you do any of the following on a regular basis to **control your salt intake**?<br>*(RECORD FOR EACH)*		
Limit consumption of processed foods	Yes 1<br>No 2	D11a
Look at the salt or sodium content on food labels	Yes 1<br>No 2	D11b
Buy low salt/sodium alternatives	Yes 1<br>No 2	D11c
Use spices other than salt when cooking	Yes 1<br>No 2	D11d
Avoid eating foods prepared outside of a home	Yes 1<br>No 2	D11e
Do other things specifically to control your salt intake	Yes 1 *If Yes, go to D11other*<br>No 2	D11f
Other (please specify)	└─┴─┴─┴─┴─┴─┴─┘	D11other
The next questions ask about the oil or fat that is most often used for meal preparation in your household, and about meals that you eat outside a home.		
What type of **oil or fat is most often** used for meal preparation in your household?<br><br>*(USE SHOWCARD)*<br>*(SELECT ONLY ONE)*	Vegetable oil 1<br>Lard or suet 2<br>Butter or ghee 3<br>Margarine 4<br>Other 5 *If Other, go to D12 other*<br>None in particular 6<br>None used 7<br>Don't know 77	D12
	Other └─┴─┴─┴─┴─┴─┴─┘	D12other
On average, how many meals per week do you eat that were not prepared at a home? By meal, I mean breakfast, lunch and dinner.	Number<br>Don't know 77 └─┴─┘	D13

CORE: Physical Activity

Next I am going to ask you about the time you spend doing different types of physical activity in a typical week. Please answer these questions even if you do not consider yourself to be a physically active person.
Think first about the time you spend doing work. Think of work as the things that you have to do such as paid or unpaid work, study/training, household chores, harvesting food/crops, fishing or hunting for food, seeking employment. *[Insert other examples if needed].* In answering the following questions 'vigorous-intensity activities' are activities that require hard physical effort and cause large increases in breathing or heart rate, 'moderate-intensity activities' are activities that require moderate physical effort and cause small increases in breathing or heart rate.

Question	Response	Code
**Work**		
Does your work involve vigorous-intensity activity that causes large increases in breathing or heart rate like *[carrying or lifting heavy loads, digging or construction work]* for at least 10 minutes continuously?<br>*[INSERT EXAMPLES] (USE SHOWCARD)*	Yes 1<br>No 2 *If No, go to P 4*	P1
In a typical week, on how many days do you do vigorous-intensity activities as part of your work?	Number of days └─┘	P2
How much time do you spend doing vigorous-intensity activities at work on a typical day?	Hours : minutes └─┴─┘ : └─┴─┘<br>hrs mins	P3 (a-b)
Does your work involve moderate-intensity activity, that causes small increases in breathing or heart rate such as brisk walking *[or carrying light loads]* for at least 10 minutes continuously?<br>*[INSERT EXAMPLES] (USE SHOWCARD)*	Yes 1<br>No 2 *If No, go to P 7*	P4
In a typical week, on how many days do you do moderate-intensity activities as part of your work?	Number of days └─┘	P5
How much time do you spend doing moderate-intensity activities at work on a typical day?	Hours : minutes └─┴─┘ : └─┴─┘<br>hrs mins	P6 (a-b)
**Travel to and from places**		
The next questions exclude the physical activities at work that you have already mentioned.<br>Now I would like to ask you about the usual way you travel to and from places. For example to work, for shopping, to market, to place of worship. *[Insert other examples if needed]*		
Do you walk or use a bicycle *(pedal cycle)* for at least 10 minutes continuously to get to and from places?	Yes 1<br>No 2 *If No, go to P 10*	P7
In a typical week, on how many days do you walk or bicycle for at least 10 minutes continuously to get to and from places?	Number of days └─┘	P8
How much time do you spend walking or bicycling for travel on a typical day?	Hours : minutes └─┴─┘ : └─┴─┘<br>hrs mins	P9 (a-b)

CORE: Physical Activity, Continued

Question	Response	Code
**Recreational activities**		
The next questions exclude the work and transport activities that you have already mentioned.<br>Now I would like to ask you about sports, fitness and recreational activities (leisure), *[Insert relevant terms]*.		
Do you do any vigorous-intensity sports, fitness or recreational *(leisure)* activities that cause large increases in breathing or heart rate like *[running or football]* for at least 10 minutes continuously?<br>*[INSERT EXAMPLES] (USE SHOWCARD)*	Yes 1<br>No 2 *If No, go to P 13*	P10
In a typical week, on how many days do you do vigorous-intensity sports, fitness or recreational *(leisure)* activities?	Number of days └─┘	P11
How much time do you spend doing vigorous-intensity sports, fitness or recreational activities on a typical day?	Hours : minutes └─┴─┘ : └─┴─┘ hrs mins	P12 (a-b)
Do you do any moderate-intensity sports, fitness or recreational *(leisure)* activities that cause a small increase in breathing or heart rate such as brisk walking, *[cycling, swimming, volleyball]* for at least 10 minutes continuously?<br>*[INSERT EXAMPLES] (USE SHOWCARD)*	Yes 1<br>No 2 *If No, go to P16*	P13
In a typical week, on how many days do you do moderate-intensity sports, fitness or recreational *(leisure)* activities?	Number of days └─┘	P14
How much time do you spend doing moderate-intensity sports, fitness or recreational *(leisure)* activities on a typical day?	Hours : minutes └─┴─┘ : └─┴─┘ hrs mins	P15 (a-b)

EXPANDED: Physical Activity

**Sedentary behaviour**		
The following question is about sitting or reclining at work, at home, getting to and from places, or with friends including time spent sitting at a desk, sitting with friends, traveling in car, bus, train, reading, playing cards or watching television, but do not include time spent sleeping.<br>*[INSERT EXAMPLES] (USE SHOWCARD)*		
How much time do you usually spend sitting or reclining on a typical day?	Hours : minutes └─┴─┘ : └─┴─┘ hrs mins	P16 (a-b)

CORE: History of Raised Blood Pressure

Question	Response	Code
Have you ever had your blood pressure measured by a doctor or other health worker?	Yes 1<br>No 2 *If No, go to H6*	H1
Have you ever been told by a doctor or other health worker that you have raised blood pressure or hypertension?	Yes 1<br>No 2 *If No, go to H6*	H2a
Have you been told in the past 12 months?	Yes 1<br>No 2	H2b
In the past two weeks, have you taken any drugs (medication) for raised blood pressure prescribed by a doctor or other health worker?	Yes 1<br>No 2	H3
Have you ever seen a traditional healer for raised blood pressure or hypertension?	Yes 1<br>No 2	H4
Are you currently taking any herbal or traditional remedy for your raised blood pressure?	Yes 1<br>No 2	H5

CORE: History of Diabetes

Have you ever had your blood sugar measured by a doctor or other health worker?	Yes 1<br>No 2 *If No, go to H12*	H6
Have you ever been told by a doctor or other health worker that you have raised blood sugar or diabetes?	Yes 1<br>No 2 *If No, go to H12*	H7a
Have you been told in the past 12 months?	Yes 1<br>No 2	H7b
In the past two weeks, have you taken any drugs (medication) for diabetes prescribed by a doctor or other health worker?	Yes 1<br>No 2	H8
Are you currently taking insulin for diabetes prescribed by a doctor or other health worker?	Yes 1<br>No 2	H9
Have you ever seen a traditional healer for diabetes or raised blood sugar?	Yes 1<br>No 2	H10
Are you currently taking any herbal or traditional remedy for your diabetes?	Yes 1<br>No 2	H11

CORE: History of Raised Total Cholesterol

Question	Response	Code
Have you ever had your cholesterol (fat levels in your blood) measured by a doctor or other health worker?	Yes 1<br>No 2 *If No, go to H17*	H12
Have you ever been told by a doctor or other health worker that you have raised cholesterol?	Yes 1<br>No 2 *If No, go to H17*	H13a
Have you been told in the past 12 months?	Yes 1<br>No 2	H13b
In the past two weeks, have you taken any oral treatment (medication) for raised total cholesterol prescribed by a doctor or other health worker?	Yes 1<br>No 2	H14
Have you ever seen a traditional healer for raised cholesterol?	Yes 1<br>No 2	H15
Are you currently taking any herbal or traditional remedy for your raised cholesterol?	Yes 1<br>No 2	H16

CORE: History of Cardiovascular Diseases

Have you ever had a heart attack or chest pain from heart disease (angina) or a stroke (cerebrovascular accident or incident)?	Yes 1<br>No 2	H17
Are you currently taking aspirin regularly to prevent or treat heart disease?	Yes 1<br>No 2	H18
Are you currently taking statins (Lovastatin/Simvastatin/Atorvastatin or any other statin) regularly to prevent or treat heart disease?	Yes 1<br>No 2	H19

CORE: Lifestyle Advice

During the past three years, has a doctor or other health worker advised you to do any of the following?
(RECORD FOR EACH)

Quit using tobacco or don't start	Yes 1<br>No 2	H20a
Reduce salt in your diet	Yes 1<br>No 2	H20b
Eat at least five servings of fruit and/or vegetables each day	Yes 1<br>No 2	H20c
Reduce fat in your diet	Yes 1<br>No 2	H20d
Start or do more physical activity	Yes 1<br>No 2	H20e
Maintain a healthy body weight or lose weight	Yes 1 *If C1=1 go to M1*<br>No 2 *If C1=1 go to M1*	H20f

CORE (for women only): Cervical Cancer Screening

The next question asks about cervical cancer prevention. Screening tests for cervical cancer prevention can be done in different ways, including Visual Inspection with Acetic Acid/vinegar (VIA), pap smear and Human Papillomavirus (HPV) test. VIA is an inspection of the surface of the uterine cervix after acetic acid (or vinegar) has been applied to it. For both pap smear and HPV test, a doctor or nurse uses a swab to wipe from inside your vagina, take a sample and send it to a laboratory. It is even possible that you were given the swab yourself and asked to swab the inside of your vagina. The laboratory checks for abnormal cell changes if a pap smear is done, and for the HP virus if an HPV test is done.

Question	Response	Code
Have you ever had a screening test for cervical cancer, using any of these methods described above?	Yes 1<br>No 2<br>Don't know 77	CX1

Participant Identification Number └─┴─┴─┘ └─┴─┴─┘ └─┴─┴─┘

Step 2 Physical Measurements

CORE: Blood Pressure		
**Question**	**Response**	**Code**
Interviewer ID	└─┴─┴─┘	M1
Device ID for blood pressure	└─┴─┘	M2
Cuff size used	Small 1<br>Medium 2<br>Large 3	M3
Reading 1	Systolic (mmHg) └─┴─┴─┘	M4a
	Diastolic (mmHg) └─┴─┴─┘	M4b
Reading 2	Systolic (mmHg) └─┴─┴─┘	M5a
	Diastolic (mmHg) └─┴─┴─┘	M5b
Reading 3	Systolic (mmHg) └─┴─┴─┘	M6a
	Diastolic (mmHg) └─┴─┴─┘	M6b
During the past two weeks, have you been treated for raised blood pressure with drugs (medication) prescribed by a doctor or other health worker?	Yes 1<br>No 2	M7
**CORE: Height and Weight**		
**For women:** Are you pregnant?	Yes 1 *If Yes, go to M 16*<br>No 2	M8
Interviewer ID	└─┴─┴─┘	M9
Device IDs for height and weight	Height └─┴─┘<br>Weight └─┴─┘	M10a<br>M10b
Height	in Centimetres (cm) └─┴─┴─┘.└─┘	M11
Weight<br>*If too large for scale 666.6*	in Kilograms (kg) └─┴─┴─┘.└─┘	M12
**CORE: Waist**		
Device ID for waist	└─┴─┘	M13
Waist circumference	in Centimetres (cm) └─┴─┴─┘.└─┘	M14

EXPANDED: Hip Circumference and Heart Rate		
Hip circumference	in Centimeters (cm) └─┴─┴─┘.└─┘	M15
Heart Rate		
Reading 1	Beats per minute └─┴─┴─┘	M16a
Reading 2	Beats per minute └─┴─┴─┘	M16b
Reading 3	Beats per minute └─┴─┴─┘	M16c

Participant Identification Number |__|__|__| |__|__|__| |__|__|__|

Step 3 Biochemical Measurements

CORE: Blood Glucose		
**Question**	**Response**	**Code**
During the past 12 hours have you had anything to eat or drink, other than water?	Yes 1<br>No 2	B1
Technician ID	|__|__|__|	B2
Device ID	|__|__|	B3
Time of day blood specimen taken (24 hour clock)	Hours : minutes |__|__| : |__|__| hrs mins	B4
Fasting blood glucose<br>*[CHOOSE ACCORDINGLY: MMOL/L OR MG/DL]*	mmol/l |__|__| . |__|__| mg/dl |__|__|__| . |__|	B5
Today, have you taken insulin or other drugs (medication) that have been prescribed by a doctor or other health worker for raised blood glucose?	Yes 1<br>No 2	B6
**CORE: Blood Lipids**		
Device ID	|__|__|	B7
Total cholesterol<br>*[CHOOSE ACCORDINGLY: MMOL/L OR MG/DL]*	mmol/l |__|__| . |__|__| mg/dl |__|__|__| . |__|	B8
During the past two weeks, have you been treated for raised cholesterol with drugs (medication) prescribed by a doctor or other health worker?	Yes 1<br>No 2	B9
**CORE: Urinary sodium and creatinine**		
Had you been fasting prior to the urine collection?	Yes 1<br>No 2	B10
Technician ID	|__|__|__|	B11
Device ID	|__|__|	B12
Time of day urine sample taken (24 hour clock)	Hours : minutes |__|__| : |__|__| hrs mins	B13
Urinary sodium	mmol/l |__|__|__| . |__|	B14
Urinary creatinine	mmol/l |__|__| . |__|__|	B15

EXPANDED: Triglycerides and HDL Cholesterol		
**Question**	**Response**	**Code**
Triglycerides<br>*[CHOOSE ACCORDINGLY: MMOL/L OR MG/DL]*	mmol/l |__|__| . |__|__| mg/dl |__|__|__| . |__|	B16
HDL Cholesterol<br>*[CHOOSE ACCORDINGLY: MMOL/L OR MG/DL]*	mmol/l |__| . |__|__| mg/dl |__|__|__| . |__|	B17
